# Size resolved characteristics of urban and suburban bacterial bioaerosols in Japan as assessed by 16S rRNA amplicon sequencing

**DOI:** 10.1038/s41598-020-68933-z

**Published:** 2020-07-22

**Authors:** Daisuke Tanaka, So Fujiyoshi, Fumito Maruyama, Motoshi Goto, Shinichi Koyama, Jun-ichi Kanatani, Junko Isobe, Masanori Watahiki, Akihiro Sakatoku, Shigehiro Kagaya, Shogo Nakamura

**Affiliations:** 1 Graduate School of Science and Engineering, University of Toyama, Toyama, Japan; 2 JST/JICA, Science and Technology Research Partnership for Sustainable Development Program (SATREPS), Tokyo, Japan; 3 Office of Academic Research and Industry-Government Collaboration, Hiroshima University, Hiroshima, Japan; 4 Murata Keisokuki Service Co., Ltd., Yokohama, Japan; 5 Department of Bacteriology, Toyama Institute of Health, Toyama, Japan

**Keywords:** Risk factors, Environmental impact

## Abstract

To study the size-resolved characteristics of airborne bacterial community composition, diversity, and abundance, outdoor aerosol samples were analysed by 16S rRNA gene-targeted quantitative PCR and amplicon sequencing with Illumina MiSeq. The samples were collected using size-resolved samplers between August and October 2016, at a suburban site in Toyama City and an urban site in Yokohama City, Japan. The bacterial communities were found to be dominated by Actinobacteria, Firmicutes, and Proteobacteria. At the genus level, we found a high abundance of human skin-associated bacteria, such as *Propionibacterium*, *Staphylococcus*, and *Corynebacterium*, in the urban site. Whereas, a high abundance of bacteria associated with soil and plants, such as *Methylobacterium* and *Sphingomonas*, was observed in the suburban site. Furthermore, our data revealed a shift in the bacterial community structure, diversity, and abundance of total bacteria at a threshold of 1.1-µm diameter. Interestingly, we observed that *Legionella* spp., the causal agents of legionellosis in humans, were mainly detected in > 2.1 µm coarse particles. Our data indicate that local environmental factors including built environments could influence the outdoor airborne bacterial community at each site. These results provide a basis for understanding the size-resolved properties of bacterial community composition, diversity, and abundance in outdoor aerosol samples and their potential influence on human health.

## Introduction

Airborne microbes can cause adverse effects for human and animals both in indoor and outdoor environments^1–3^. Aerosols of biological origin (bioaerosols) originate from different natural and anthropogenic activities with different emission characteristics for every source. Bioaerosols are emitted from soil, freshwater, ocean, vegetation, animal feces, human skin, oral cavity, wastewater treatment plant, and composting facilities^4–6^. It has been estimated that about 25% of atmospheric aerosol particles is from biological sources^5^. A better understanding of the composition and concentration of bioaerosols is needed to minimize their impact on human health.

Airborne microbes are ubiquitous in the air and mainly include bacteria, fungi, viruses, pollen, and archaea^7,8^. Bacteria associated with particulate matter (PM) are present in the atmosphere in the form of spores, vegetative cells, or dividing cells^9,10^. Particle size is an important factor for the degree of inhalability of PM, which has a crucial effect on human health. For example, coarse particles are mainly deposited in the extra thoracic region, whereas fine particles have a higher probability of being deposit deeper in the trachea, bronchial and alveolar regions^11^. Therefore, PM size distribution is a significant factor when researching the health risk of exposure to airborne bacteria in humans. However, there are insufficient studies of bacterial diversity and abundance in size-resolved aerosol samples using molecular biology methods^12–14^.

Spatio-temporal variability of microbial communities is an important factor that provides insight into atmospheric biodiversity and biogeography^15–17^. In this study, we monitored the bacterial abundance and community composition of outdoor aerosol samples collected using size-resolved samplers at a suburban site in Toyama City and an urban site in Yokohama City, Japan. The elevation and distance from the sea are remarkably similar between the two locations. To study the size-resolved characteristics of airborne bacterial community composition, diversity, and abundance, we used quantitative PCR and Illumina MiSeq sequencing.

## Results

### Sequencing

We obtained 2,291,974 raw sequence reads from 54 samples collected at a suburban site in Toyama City and an urban site in Yokohama City, Japan (Supplementary Fig. S1, Supplementary Table S1 and S2). A total of 1,647,648 reads (30,512 reads per sample) were clustered into 1,158 operational taxonomic units (OTUs) (97% similarity). Good's coverage values were greater than 99% for all samples.

### Bacterial community composition

The bacterial community was dominated by three phyla: Proteobacteria (45.1%), Actinobacteria (24.4%), and Firmicutes (18.6%) (Supplementary Fig. S2). At the class level, the dominant groups were Actinobacteria (23.6%), Alphaproteobacteria (23.3%), Bacilli (16.9%), Gammaproteobacteria (14.4%), and Betaproteobacteria (6.0%). At the genus level, the dominant groups included *Staphylococcus* (8.9%), *Propionibacterium* (7.5%), *Corynebacterium* (5.9%), *Sphingomonas* (5.0%), *Methylobacterium* (4.6%), and *Streptococcus* (3.7%).

### Comparison of bacterial community structure

**Figure 1:**
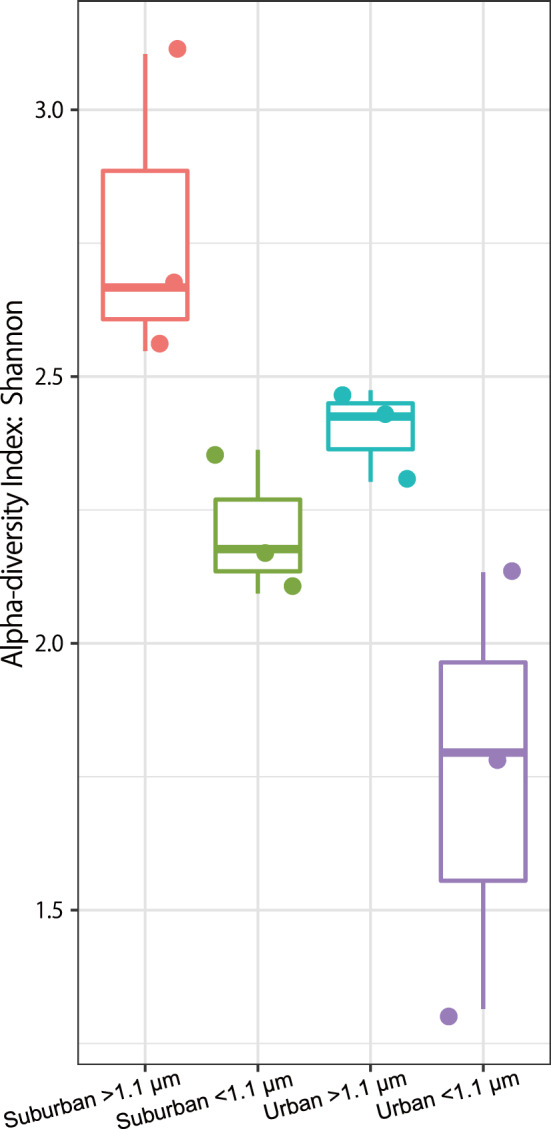
Comparison of the bacterial diversity indexes in air samples from the suburban (> 1.1 µm, red), suburban (< 1.1 µm, green), urban (> 1.1 µm, blue), and urban (< 1.1 µm, purple) groups. Box plots represent (from top to bottom) maximum, upper-quartile, median, lower-quartile, and minimum values. The median in three points (August, September, October) is shown for each of the four sample groups.

**Figure 2:**
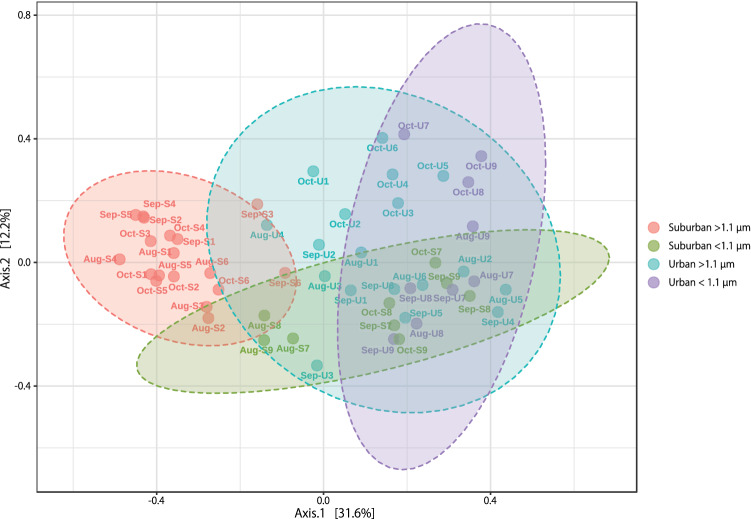
Principal coordinates plot showing the overall variation in bacterial communities. Communities are compared using the Bray–Curtis distance metric. Fifty-four samples from the suburban (> 1.1 µm, red), suburban (< 1.1 µm, green), urban (> 1.1 µm, blue), and urban (< 1.1 µm, purple) were plotted with two coordinates. The mean and standard deviation for each axis are indicated by an ellipse for each sample group. Difference among four sample groups are significant (PERMANOVA, R = 0.313, p < 0.001).

**Figure 3:**
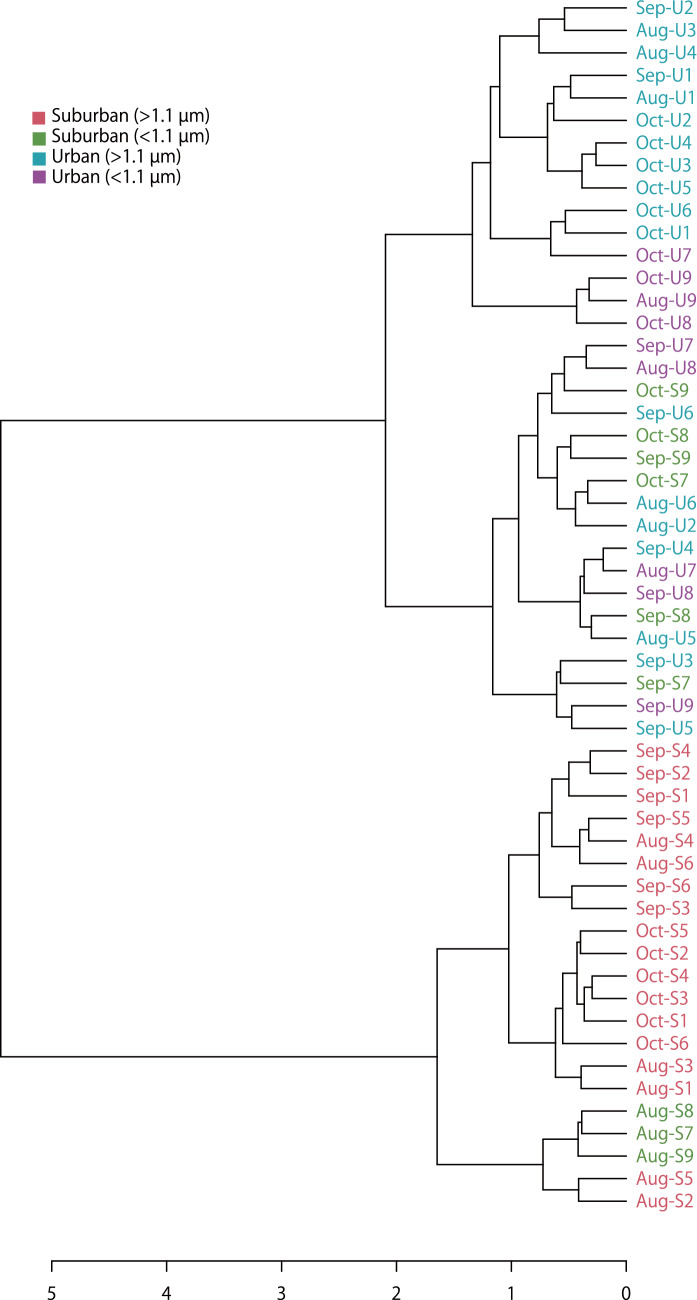
Hierarchical clustering of the bacterial community based on distance measurement using Bray–Curtis dissimilarity index and clustering algorithm using Ward distance. Air samples from suburban (> 1.1 µm, red, n = 18) grouped separately from those from suburban (< 1.1 µm, green, n = 9) and urban (> 1.1 µm, blue, n = 18, and < 1.1 µm, purple, n = 9) in most cases. The bacterial communities detected in the suburban (< 1.1 µm) and urban (> 1.1 µm, < 1.1 µm) groups could not be readily distinguished.

**Figure 4:**
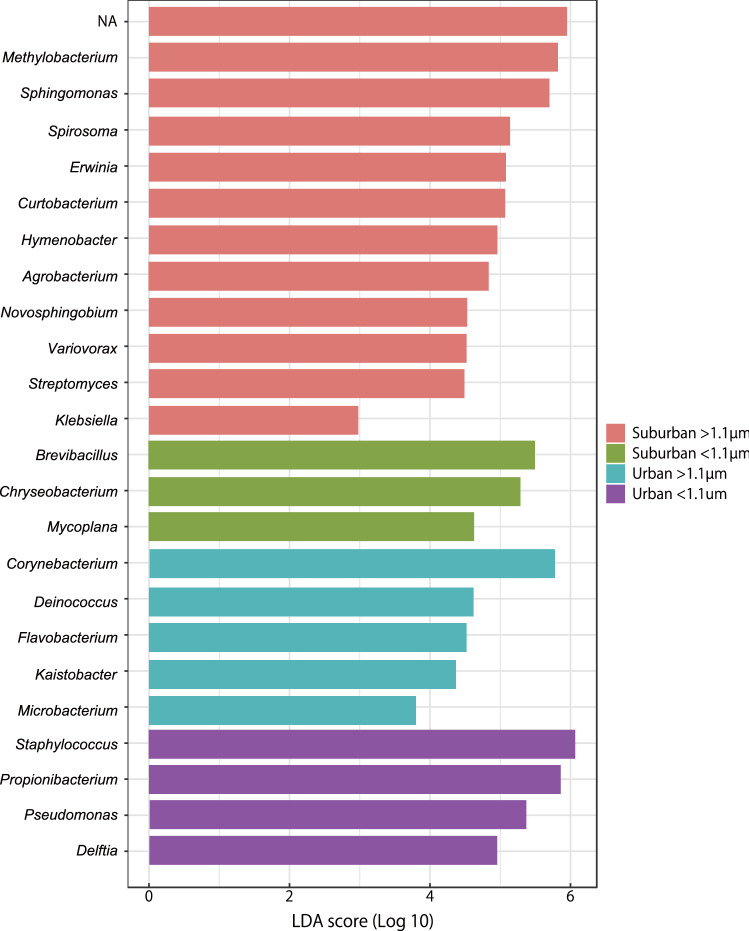
The LEfSe identified the most differentially abundant genera within the suburban (> 1.1 µm, red), suburban (< 1.1 µm, green), urban (> 1.1 µm, blue), and urban (< 1.1 µm, purple) groups. NA, not assigned. Totally 23 bacterial genera were considered significant. The threshold for the logarithmic LDA score is 2, with a p-value of < 0.05.

The Shannon alpha-diversity indexes of bacteria associated with the suburban samples were greater than those associated with the urban samples at both > 1.1 µm and < 1.1 µm (Fig. 1). A principal coordinates plot and hierarchical clustering of the bacterial community showed that air samples from suburban (> 1.1 µm; red colour) grouped separately from those of other sample groups in most cases (Figs. 2, 3). Linear discriminant analysis (LDA) effect size (LEfSe) analysis revealed 23 genera with an LDA score of at least 2.0 that were significantly more abundant in the four sample groups (Fig. 4). Specifically, we found five genera to be enriched in the samples: *Staphylococcus* and *Propionibacterium* in the urban samples (< 1.1 µm; purple colour), *Corynebacterium* in the urban samples (> 1.1 µm; blue colour), and *Methylobacterium* and *Sphingomonas* in the suburban samples (> 1.1 µm; red colour). All of these were fairly abundant in the samples (at least 4% of the population).

### Comparison of bacterial abundance

The total bacterial gene copy number in samples > 1.1 µm was 3.1-fold higher than in samples < 1.1 µm (Supplementary Fig. S3). The abundance of total bacteria in samples of > 1.1 µm ranged from 1.3 × 10^3^ to 5.0 × 10^4^ copies m^−3^ (1.5 × 10^4^ copies m^−3^ on average). Whereas, the abundance of total bacteria in samples of < 1.1 µm ranged from 1.0 × 10^3^ to 1.7 × 10^4^ copies m^−3^ (5.0 × 10^3^ copies m^−3^ on average). From the perspective of the sampling area, the total bacterial gene copy number in the suburban samples was 1.7-fold higher than in the urban samples (Supplementary Fig. S3). The abundance of total bacteria in the suburban samples ranged from 1.6 × 10^3^ to 5.0 × 10^4^ copies m^−3^ (1.5 × 10^4^ copies m^−3^ on average). Whereas, the abundance of total bacteria in the urban samples ranged from 1.0 × 10^3^ to 3.7 × 10^4^ copies m^−3^ (8.9 × 10^3^ copies m^−3^ on average).

### *Legionella*-assigned OTUs

**Figure 5:**
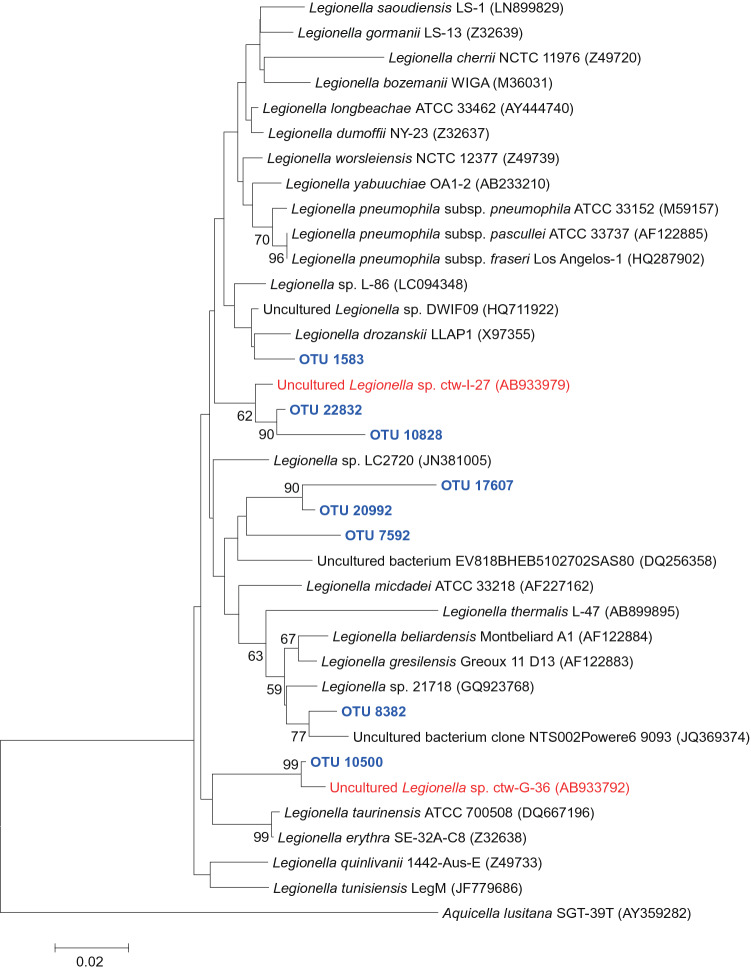
Neighbor-joining phylogenetic tree based on partial 16S rRNA gene sequences illustrating the phylogenetic affiliation of the assigned OTUs (blue colour) within the genus *Legionella*. The 1,000 resampling bootstrap values (%) are indicated at the nodes; only values greater than 50 are shown. *Aquicella lusitana* is used as an outgroup. The scale bar represents 0.02 substitutions per base position. The sequences derived from cooling tower water in Japan^18^ are shown in red colour.

Interestingly, the bacterial pathogen *Legionella* spp. was detected. The results provide valuable data for hazard evaluation of the effects of bioaerosols on human health. We examined *Legionella*-assigned OTUs in air samples (Fig. 5, Supplementary Table S3). Among nine samples, *Legionella* spp. were mainly detected in coarse particle samples (> 2.1 µm; Stage 1 to 5 of size-resolved sampler). The detection rate of the *Legionella*-assigned OTUs ranged from 0.004 to 1.421% (0.479% on average). Phylogenetic analysis of the genus *Legionellae* showed that some OTUs were closely related to the sequences from cooling tower water samples in Japan^18^.

## Discussion

In the present study, we examined the bacterial community composition and diversity of outdoor aerosol samples using size-resolved samplers and Illumina MiSeq sequencing at a suburban site in Toyama City and an urban site in Yokohama City, Japan. At any point in time, different sites have their own unique bacterial communities, and local sources of bacteria and other environmental factors may be involved in the assembly of the communities. Bacterial composition, diversity, abundance, and predominant genera showed size-resolved characteristics.

The bacterial composition was congruent with those reported in other bioaerosol studies: with the airborne bacterial community dominated by Proteobacteria, Actinobacteria, and Firmicutes at the phylum level, and Actinobacteria, Alphaproteobacteria, Bacilli, Gammaproteobacteria, and Betaproteobacteria at the class level^16,17,19–22^.

As shown in Fig. 4, LEfSe analysis showed that the high abundance of human skin-associated bacteria, such as *Propionibacterium, Staphylococcus*, and *Corynebacterium*, may be a feature of urban site^23,24^. This situation could indicate that outdoor bacterial communities were influenced by bacteria in the built environment. Whereas, the high abundance of bacteria associated with soil and plants, such as *Methylobacterium* and *Sphingomonas*, may be a feature of suburban site^24^. These five bacterial genera have been frequently detected in outdoor and indoor air^23–25^. The genus *Propionibacterium* is a Gram-positive, anaerobic, rod-shaped bacteria that produces propionic acid as its end product of fermentation. This genus in the family *Propionibacteriaceae* consists of species from various habitats, including mature cheese, cattle rumen, and human skin^26^. The commensal bacterium *Cutibacterium acnes* (formerly *Propionibacterium acnes*) is involved in the maintenance of human skin, and it is also the pathogen responsible for acne vulgaris and other diseases^27^. The genus *Staphylococcus* comprises Gram-positive, spherical (cocci) bacteria that form grape-like clusters and are facultative anaerobes. Most are harmless and reside normally on the skin and mucous membranes of humans and other organisms. In humans, *S. epidermidis* is the most frequently recovered staphylococcal species^28^. The genus *Corynebacterium* comprises Gram-positive, non-sporulating (although they have club-like ends), aerobic, pleomorphic bacilli that are isolated from a range of environments including soil, water, blood, and human skin. Pathogenic strains of *Corynebacterium* can infect animals or humans^29,30^. The genus *Methylobacterium* comprises Gram-negative, obligately aerobic, rod-shaped bacteria. This genus displays pink pigmentation and the bacteria are facultative methylotrophs. They are ubiquitous in nature and are commonly found in the atmosphere, soil, water, and in the phylloplane, where some may produce plant growth-promoting substances^31^. Rarely, *Methylobacterium* species are found in clinical samples as opportunist pathogens. The genus *Sphingomonas* comprises Gram-negative, non-spore-forming, chemoheterotrophic, strictly aerobic bacteria. This genus typically produces yellow-pigmented colonies and has been detected in various environments, including soil, water, clinical specimens, the plant phyllosphere and rhizosphere, air, and other locations^32^.

Airborne bacteria vary in size from 0.1 to 5.0 µm^33,34^. Smaller sized bacteria more easily attach to fine particles such as PM_2.5_, or even smaller particles^15^. It has been suggested that the bacterial concentration and size distribution vary with sampling site. Interestingly, our data revealed a shift in the bacterial community structure, diversity, and abundance in PM at a threshold of 1.1 µm diameter in size. Similarly, Wei et al*.*^35^ reported a disparity in bacterial communities according to the abundance of rare species, such as *Bacilli* being higher in PM_1.0_ (2.4%) than in PM_2.5_ (1.8%), and *Defluviicoccus* being higher in PM_2.5_ (2.5%) than in PM_1.0_ (0.5%), which may be associated with cell size and cell growth patterns. Blais Lecours et al*.*^36^ showed that bacteria mainly attached to particles with a diameter larger than 2.1 µm. Further studies are needed to evaluate the size-resolved characteristics of airborne bacterial communities.

Interestingly, we observed that *Legionella* spp., the causal agents of legionellosis including a pneumonia-type illness known as Legionnaires' disease and a mild flu-like illness known as Pontiac fever, were mainly detected in > 2.1-µm coarse particles. Assuming the average of rRNA gene copy number of 4 per bacterial genome and 3 per *Legionella* genome (https://rrndb.umms.med.umich.edu/), our results suggest that *Legionella* spp. represented less than 1% of the total bacterial community. Mathieu et al*.*^37^ observed that *Legionella* bacteria represented a small fraction 0.05–0.9% of the total airborne biocontaminants above the fan of the cooling towers and close to an industrial sludge water treatment basin. To our knowledge, this is the first study to detect *Legionella* spp. in outdoor air samples in Japan. In addition, Fig. 5 shows that some OTUs were closely related to the sequences from cooling tower water samples in Japan^18^. Therefore, we hypothesized that outdoor aerosol samples in Japan often contain *Legionella* derived from cooling tower water. However, the short fragments generated by 16S rRNA amplicon sequencing on the illumina miseq platform (~ 400 bp in this study) limit their use for 16S rRNA gene-based bacterial identification. Currently, the genus *Legionella* comprises more than 60 different bacterial species and 70 serogroups that live in many environments, both natural (e.g., rivers, lakes, soil, and ponds) and artificial (e.g., swimming pools, showers, cooling towers, fountains, and waste water treatment plants). Within the genus *Legionella*, several species can cause clinical disease in humans, such as *L. pneumophila*, *L. dumoffii, L. bozemanii*, *L. longbeachae*, and *L. micdadei*^38,39^. *Legionella pneumophila* serogroup 1 is the most virulent strain causing the vast majority of *Legionella* infections. In the present study, common causative agents of legionellosis were not detected. However, all species of the genus *Legionella* are potentially pathogenic in humans^40^. According to the nationwide sentinel surveillance system^41^, the peak season for legionellosis was July in Japan; although more patients were reported from more populated prefectures as expected, the number of patients per 100,000 population was high in Toyama, Ishikawa, Okayama, and Tottori Prefectures. Kanatani et al*.*^42^ found that puddles on asphalt roads could serve reservoirs for *L. pneumophila* in the environment, which can increase potential opportunities for exposure.

In conclusion, this study provides useful information on the size-resolved bacterial communities in outdoor aerosol samples. The results showed that size-resolved differences occurred in terms of airborne bacterial community composition, diversity, and abundance at a suburban site in Toyama City and an urban site in Yokohama City, Japan. The most likely source of airborne bacteria in the urban site was humans. Furthermore, we detected *Legionella* spp., the causal agents of legionellosis in humans, and these organisms could often be derived from cooling tower water. These findings could provide a foundation for understanding the transmission and health effects of bioaerosols.

## Materials and methods

### Air samples

Air samples were collected between August and October 2016, in Toyama City and Yokohama City in Japan. The Toyama sampling site was in a suburban area located on the roof of the University of Toyama (36° 41′ 54′' N, 137° 11′ 13′' E, 23 m asl). The Yokohama sampling site was in an urban area located on the roof of the Murata Keisokuki Service Co. Ltd. (35° 25′ 17′' N, 139° 33′ 00′' E, ~ 20 m asl). The population of the Toyama City area is about 420,000, and that of the Yokohama City area is about 3,750,000. Yokohama is the second largest city in Japan by population, and is extremely close to Tokyo. The two sites were located about 250 km apart from one another. Meteorological data were obtained from Toyama Local Meteorological Office (https://www.jma-net.go.jp/toyama/) located approximately 1.8 km northeast of the Toyama sampling site and Yokohama Local Meteorological Office (https://www.jma-net.go.jp/yokohama/) located approximately 9.6 km east-northeast of the Yokohama sampling site (Supplementary Table S1).

We sampled onto 80 mm diameter quartz fiber filters (2500QAT-UP, Tokyo dyrec, Japan) using an nine-stage Andersen samplers (AN-200, Sibata Scientific, Tokyo, Japan) at a flow rate of 28.3 L min^−1^ over 24 h (11:00 to 11:00 the next day) according to their aerodynamic diameter: > 11.0 µm (stage 1), 7.0–11 µm (stage 2), 4.7–7.0 µm (stage 3), 3.3–4.7 µm (stage 4), 2.1–3.3 µm (stage 5), 1.1–2.1 µm (stage 6), 0.65–1.1 µm (stage 7), 0.43–0.65 µm (stage 8), < 0.43 µm (stage 9, back-up filter)^43^. Before sampling, all of the filters were baked in a muffle furnace at 350 °C for 1 h. To avoid contamination, the sampling filter holder and materials used for changing filters were treated with 100% ethanol before use. After sampling, the filters were stored at ˗20 °C until DNA extraction.

### DNA extraction and Illumina MiSeq sequencing

The filtered samples were processed using the PowerSoil DNA isolation kit (MO BIO Laboratories, Carlsbad, CA, USA) following the manufacturer’s instructions. To prevent potential contamination, DNA extraction and PCR preparation were carried out in a laminar airflow clean bench. Subsequently, the V3–V4 region of the bacterial 16S rRNA gene was amplified using primers 1st-341F (5′-ACACTCTTTCCCTACACGACGCTCTTCCGATCT-CCTACGGGNGGCWGCAG-3′) and 1st-805R (5′-GTGACTGGAGTTCAGACGTGTGCTCTTCCGATCT-GACTACHVGGGTATCTAATCC-3′)^44^. For the first PCR amplification, the initial denaturation was at 94 °C for 2 min, followed by 35 cycles of denaturation at 94 °C for 30 s, annealing at 55 °C for 30 s, and extension at 72 °C for 30 s, with a final extension at 72 °C for 5 min. The first PCR products were purified by Agencourt AMPure XP (Beckman Coulter, Brea, CA, USA). The second PCR was conducted using primers 2ndF (5′-AATGATACGGCGACCACCGAGATCTACAC-Index2-ACACTCTTTCCCTACACGACGC-3′) and 2ndR (5′-CAAGCAGAAGACGGCATACGAGAT-Index1-GTGACTGGAGTTCAGACGTGTG-3′). The index pair was specific to each sample for an accurate recognition of the samples. The second PCR cycling conditions were 94 °C for 2 min, followed by 10 cycles of 94 °C for 30 s, 60 °C for 30 s, and 72 °C for 30 s, with a final extension at 72 °C for 5 min. The second PCR products were purified by Agencourt AMPure XP. DNA quantification was carried out by Synergy H1 (Bio Tek, Tokyo, Japan) and QuantiFluor dsDNA System (Promega, Madison, WI, USA). Purified amplicons were pooled in equimolar concentrations and paired-end sequenced (2 × 300 bp) on an Illumina Miseq instrument (Illumina, San Diego, CA, USA) with the Miseq reagent kit V3 600 cycles (Illumina) according to standard protocols.

Quality filtering was performed using the Fastx toolkit version 0.0.14^45^ and sickle^46^ with a minimum Sanger quality of 20 and a minimum length of 150. Paired sequence reads were assembled using FLASH^47^ with a minimum overlap of 10. The obtained sequence data were then processed using USEARCH version 10.0.240^48^ and analysed with the software package Quantitative Insights into Microbial Ecology (QIIME) version 1.9.1^49^. Sequences were clustered into operational taxonomic units (OTUs) using the Greengenes 13_8 reference OTU database^50^ (97% similarity). For 16S rRNA gene fragment analysis, singleton, chloroplast and mitochondrial OTUs were removed. All samples were rarefied to even sequencing depth based on the sample having the lowest sequencing depth of 17,478 reads (sample: Oct-U7) before total sum normalization. Statistical analysis was conducted using the R software, version 3.5.2^51^. We used ggplot2 package^52^ and the phyloseq package^53^. Beta-diversity was explored by principal coordinate analysis (PCoA) of Bray–Curtis dissimilarity among sample groups with different locations and size. Statistical significance was calculated by the permutational multivariate analysis of variance (PERMANOVA) in vegan package^54^. LEfSe was applied to identify specific bacterial genera among sample groups^55^. Hierarchical cluster analysis was performed based on Bray–Curtis dissimilarity matrices of relative abundance of bacterial OTU with “stats” in R package^51^. Taxa were considered significant based on LDA score of > 2 and p-value < 0.05. A phylogenetic tree was constructed using the neighbour-joining method with Kimura 2 parameter distances in MEGA X software. All sequences have been deposited in the DNA Data Bank of Japan (DDBJ) under the accession number DRA009183.

### Real-time TaqMan PCR

Real-time TaqMan PCR reactions were performed using a Thermal Cycler Dice Real Time System (TP-850, Takara Bio, Otsu, Japan). Quantification of the 16S rRNA gene of the total bacteria was performed as previously described^56^. Each reaction mixture was prepared in a total volume of 25 µL with 12.5 µL Premix Ex Taq (Probe qPCR, Takara Bio), 0.2 µM forward primer 1055f, 0.2 µM reverse primer 1392r, 0.25 µM TaqMan probe 16Staq1115, and 2 µL of standard or extracted DNA. For the assay, the PCR program was 30 s at 95 °C, followed by 40 cycles of 5 s at 95 °C, and 30 s at 60 °C. DNA standards were prepared from serial dilutions of pGEM-T Easy Vector (Promega) containing the 16S rRNA gene from *Escherichia coli* K-12 strain W3110. Duplicate aliquots of the standards and the samples were included in each PCR run. All assays included a negative control in which no template was present.

## Supplementary information

Supplementary Information
