# Supplementary material for: Size resolved characteristics of urban and suburban bacterial bioaerosols in Japan as assessed by 16S rRNA amplicon sequencing
Source: Sci Rep. 2020 Jul 22;10:12406. doi: 10.1038/s41598-020-68933-z (PMC7376176; doi:10.1038/s41598-020-68933-z)
Supplement: Supplementary file 1 — Supplementary Information [file 41598_2020_68933_MOESM1_ESM.pdf]

Supplementary Information

Size resolved characteristics of urban and suburban bacterial bioaerosols in Japan as assessed by 16S rRNA amplicon sequencing

Daisuke Tanaka[1], So Fujiyoshi[2, 3], Fumito Maruyama[2, 3], Motoshi Goto[1], Shinichi Koyama[4], Jun-ichi Kanatani[5], Junko Isobe[5], Masanori Watahiki[5], Akihiro Sakatoku[1], Shigehiro Kagaya[1] & Shogo Nakamura[1]

[1]Graduate School of Science and Engineering, University of Toyama, Toyama, Japan.

[2]JST/JICA, Science and Technology Research Partnership for Sustainable Development Program (SATREPS), Tokyo, Japan.

[3]Office of Academic Research and Industry-Government Collaboration, Hiroshima University, Hiroshima, Japan.

[4]Murata Keisokuki Service Co., Ltd., Yokohama, Japan.

[5]Department of Bacteriology, Toyama Institute of Health, Toyama, Japan.

Correspondence and requests for materials should be addressed to D. Tanaka
(email: tanakada@sci.u-toyama.ac.jp)

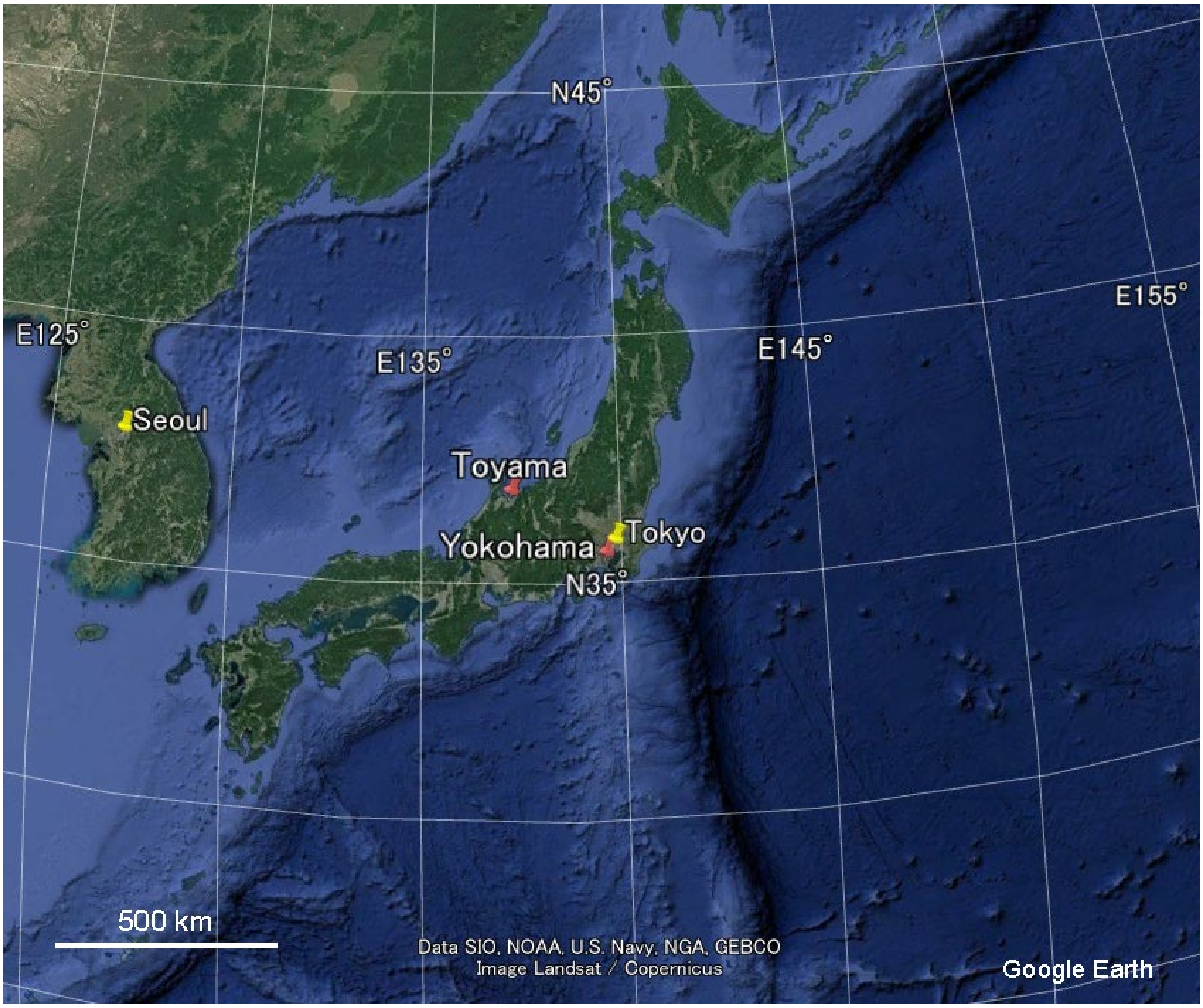

Figure S1. Map showing the sampling locations at a suburban site in Toyama City and an urban site in Yokohama City, Japan. The two sites were located about 250 km apart from one another. The sampling sites were located on the roofs of buildings.

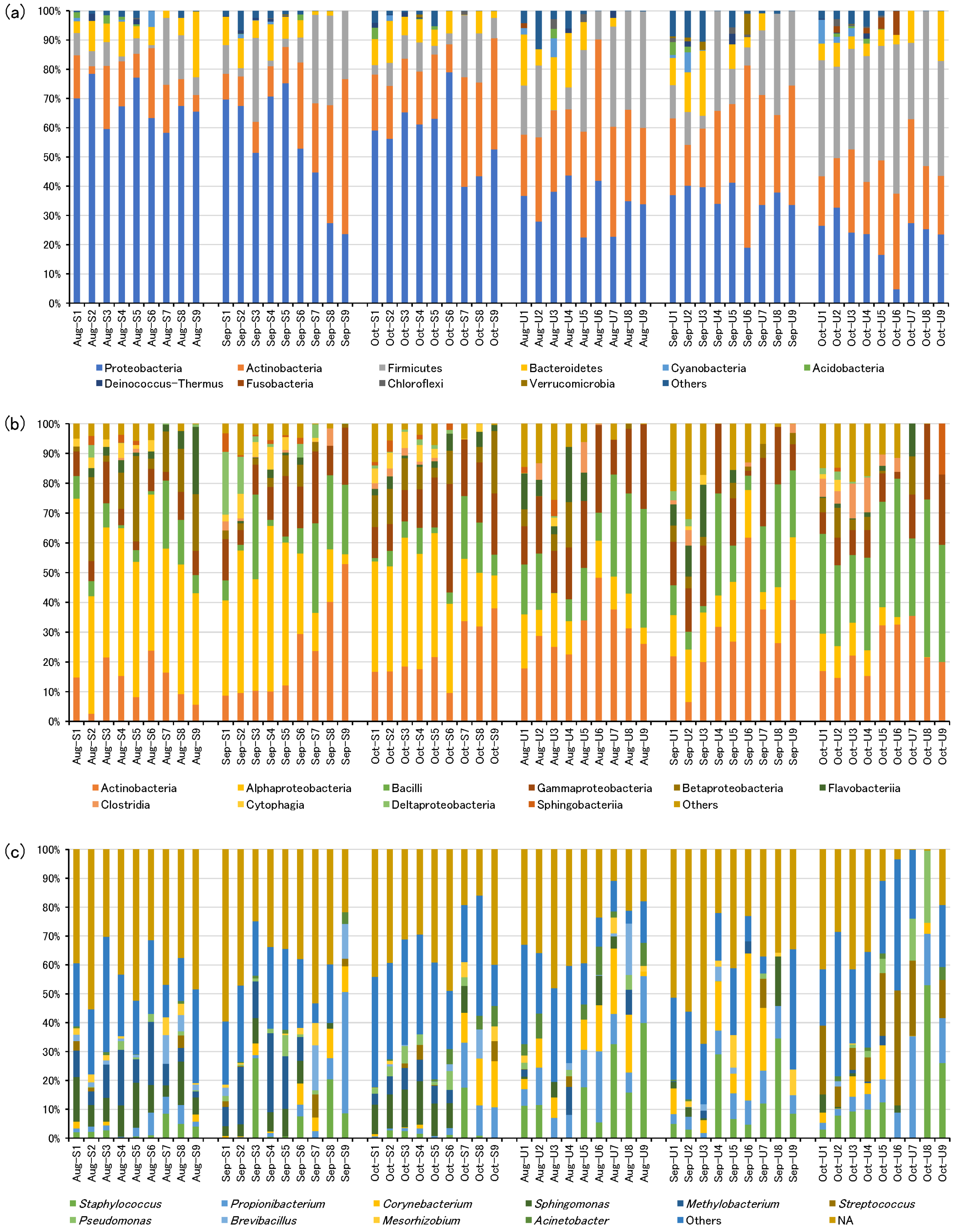

Figure S2. Taxonomic composition of bacteria at the phylum (a), class (b), and genus (c) levels in air samples collected at the suburban (S) and urban (U) sites. NA, not assigned.

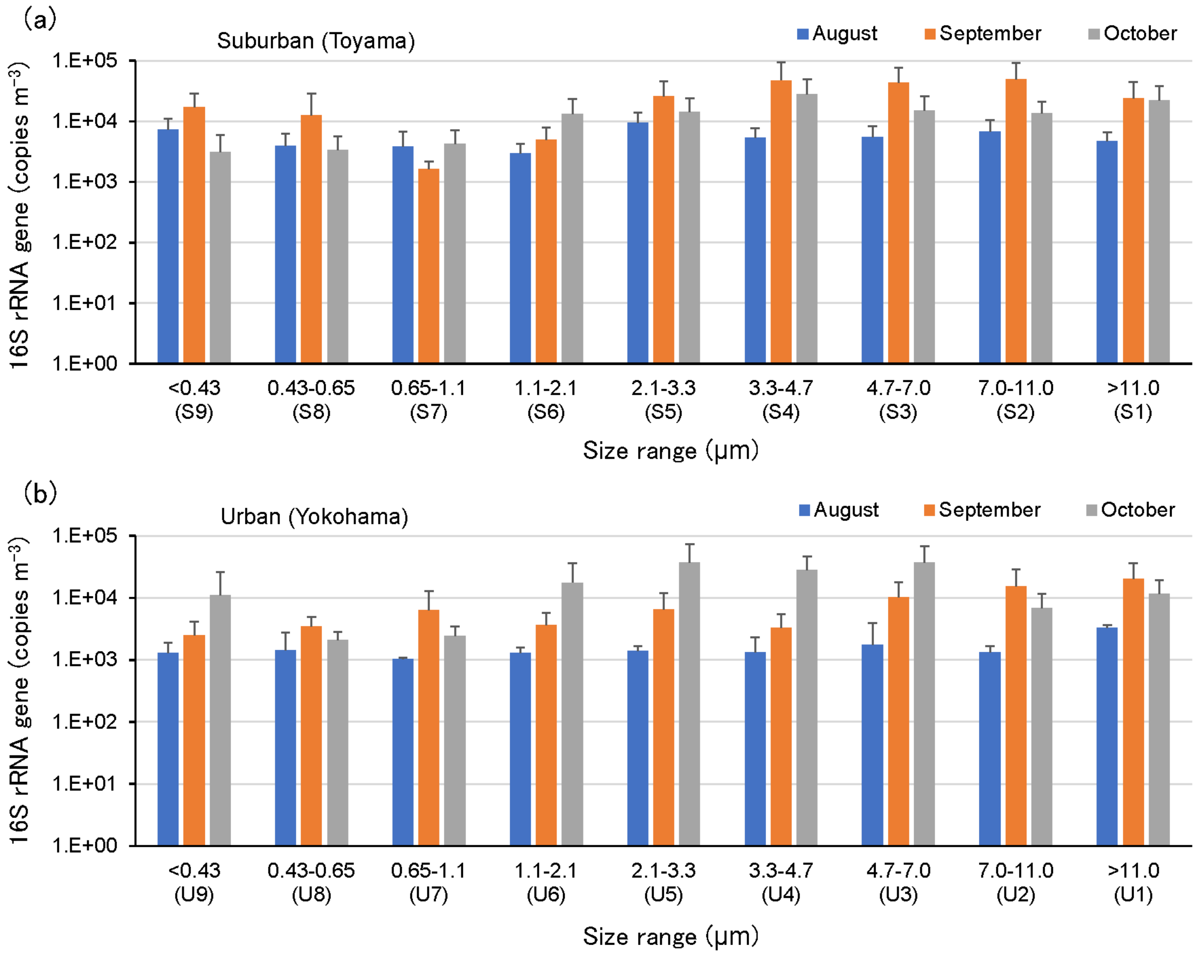

Figure S3. Quantification of the total bacterial 16S rRNA genes in air samples collected at the suburban (a) and urban (b) sites using real-time TaqMan PCR.

Table S1. Sampling information

Location	Date (2016)	Sample	Average temperature (°C)	Average relative humidity (%)	Average wind velocity (m/s)	Predominant wind direction	Precipitation (mm)
Toyama	Aug 31	Aug-S1 ~ Aug-S9	27.6	43.5	3.8	SSW	0
	Sep 27	Sep-S1 ~ Sep-S9	25.6	83.7	2.0	NNE	3
	Oct 25	Oct-S1 ~ Oct-S9	19.0	73.1	5.5	S	4.5
Yokohama	Aug 31	Aug-U1 ~ Aug-U9	27.1	65.5	5.0	SSW	0
	Sep 27	Sep-U1 ~ Sep-U9	26.7	84.8	3.1	SSW	0
	Oct 25	Oct-U1 ~ Oct-U9	16.4	84.3	2.4	N	3

Table S2. Summary of Illumina MiSeq reads

Sample	Raw sequences	Number of clean sequences	OTUs[a]	Good's coverage
Aug-S1	44,967	27,607	539	99.99%
Aug-S2	45,936	25,494	331	99.99%
Aug-S3	35,904	22,083	318	99.99%
Aug-S4	39,079	27,056	509	99.97%
Aug-S5	45,231	30,576	545	99.98%
Aug-S6	43,795	31,687	466	99.99%
Aug-S7	35,747	29,088	187	99.99%
Aug-S8	34,667	24,404	363	100.00%
Aug-S9	47,898	35,302	292	99.99%
Aug-U1	48,373	36,798	228	99.97%
Aug-U2	39,145	27,558	116	99.97%
Aug-U3	48,035	37,814	222	99.95%
Aug-U4	43,837	30,998	154	99.98%
Aug-U5	38,277	28,568	101	99.99%
Aug-U6	36,753	27,626	277	99.99%
Aug-U7	32,525	22,748	200	99.99%
Aug-U8	32,827	24,494	149	99.98%
Aug-U9	28,368	22,668	89	99.98%
Sep-S1	44,305	30,888	300	100.00%
Sep-S2	54,494	35,557	428	99.99%
Sep-S3	45,492	31,623	540	99.99%
Sep-S4	42,476	29,093	521	100.00%
Sep-S5	46,393	31,221	585	100.00%
Sep-S6	29,603	21,624	268	99.98%
Sep-S7	39,936	30,534	113	99.99%
Sep-S8	43,041	24,998	124	99.97%
Sep-S9	42,490	34,193	164	99.99%
Sep-U1	50,894	28,918	242	99.94%
Sep-U2	52,764	37,736	217	99.97%
Sep-U3	48,207	32,398	175	99.98%
Sep-U4	34,491	26,920	112	99.97%
Sep-U5	41,724	31,266	171	99.97%
Sep-U6	34,689	27,391	70	99.97%
Sep-U7	36,235	26,725	120	99.99%
Sep-U8	30,711	25,576	253	99.98%
Sep-U9	34,653	27,626	98	99.98%
Oct-S1	69,202	45,313	659	99.99%
Oct-S2	48,779	33,804	591	99.98%
Oct-S3	53,339	37,317	615	99.99%
Oct-S4	46,565	31,452	654	99.99%
Oct-S5	50,931	37,418	581	99.99%
Oct-S6	49,033	35,752	368	99.97%
Oct-S7	42,507	34,037	224	99.97%
Oct-S8	41,474	33,927	139	99.97%
Oct-S9	51,658	40,033	136	99.99%
Oct-U1	55,383	40,267	232	99.96%
Oct-U2	55,693	38,218	188	99.98%
Oct-U3	49,489	33,133	334	99.95%
Oct-U4	37,340	26,401	358	99.98%
Oct-U5	48,102	36,682	347	99.98%
Oct-U6	41,182	33,585	232	99.97%
Oct-U7	21,444	17,478	94	99.99%
Oct-U8	23,909	19,721	143	99.99%
Oct-U9	31,982	26,254	128	99.96%

[a] The operational taxonomic units (OTUs) were defined with 3 % dissimilarity.

Table S3. *Legionella* -assigned OTUs in air samples collected using size-resolved samplers

OTU ID	Sample ID									Total
	Aug-S4	Aug-S5	Aug-S8	Aug-S9	Aug-U3	Sep-U3	Oct-S5	Oct-U2	Oct-U4	
OTU 17607	1	3	0	0	0	0	0	0	0	4
OTU 20992	41	94	0	22	0	0	74	0	1	232
OTU 7592	0	4	0	1	0	0	0	0	0	5
OTU 8382	0	0	0	0	0	0	0	540	0	540
OTU 10500	0	0	0	0	0	390	0	0	0	390
OTU 22832	0	166	47	24	0	0	0	0	0	237
OTU 1583	0	3	0	0	0	0	0	0	0	3
OTU 10828	0	0	2	1	0	0	0	0	0	3
Others	6	6	5	6	2	1	7	3	0	36
Total	48	276	54	54	2	391	81	543	1	1450
Number of clean sequences	27,056	30,576	24,404	35,302	37,814	32,398	37,418	38,218	26,401	289,587
Detection rate of *Legionella* (%)	0.177%	0.903%	0.221%	0.153%	0.005%	1.207%	0.216%	1.421%	0.004%	
